# Extreme temperature and out-of-hospital-cardiac-arrest. Nationwide study in a hot climate country

**DOI:** 10.1186/s12940-021-00722-1

**Published:** 2021-04-05

**Authors:** Hannan Kranc, Victor Novack, Alexandra Shtein, Rimma Sherman, Lena Novack

**Affiliations:** 1 Department of Public Health, Faculty of Health Sciences, School of Medicine, Ben-Gurion University of the Negev, Beer Sheva, Israel; 2 Clinical Research Center, Soroka University Medical Center, Beer Sheva, Israel; 3 Department of Internal Medicine, Soroka University Medical Center, Beer Sheva, Israel; 4 Department of Geography and Environmental Development, Faculty of Humanities and Social Sciences, Ben-Gurion University of the Negev, Beer Sheva, Israel; 5 Endicott College, Beverly, MA USA; 6 Negev Environmental Health Research Institute, Soroka University Medical Center, 84101 Beer Sheva, Israel; 7 Faculty of Health Sciences, Ben-Gurion University of the Negev, Beer Sheva, Israel

**Keywords:** OHCA, Meteorology, Climate change, Temperature, Humidity, Solar radiation

## Abstract

**Background:**

Out-of-hospital-cardiac arrest (OHCA) is frequently linked to environmental exposures. Climate change and global warming phenomenon have been found related to cardiovascular morbidity, however there is no agreement on their impact on OHCA occurrence. In this nationwide analysis, we aimed to assess the incidence of the OHCA events attended by emergency medical services (EMS), in relation to meteorological conditions: temperature, humidity, heat index and solar radiation.

**Methods:**

We analyzed all adult cases of OHCA in Israel attended by EMS during 2016–2017. In the case-crossover design, we compared ambient exposure within 72 h prior to the OHCA event with exposure prior to the four control times using conditional logistic regression in a lag-distributed non-linear model.

**Results:**

There were 12,401 OHCA cases (68.3% were pronounced dead-on-scene). The patients were on average 75.5 ± 16.2 years old and 55.8% of them were males.

Exposure to 90th and 10th percentile of temperature adjusted to humidity were positively associated with the OHCA with borderline significance (Odds Ratio (OR) =1.20, 95%CI 0.97; 1.49 and OR 1.16, 95%CI 0.95; 1.41, respectively). Relative humidity below the 10th percentile was a risk factor for OHCA, independent of temperature, with borderline significance (OR = 1.16, 95%CI 0.96; 1.38). Analysis stratified by seasons revealed an adverse effect of exposure to 90th percentile of temperature when estimated in summer (OR = 3.34, 95%CI 1.90; 3.5.86) and exposure to temperatures below 10th percentile in winter (OR = 1.75, 95%CI 1.23; 2.49). Low temperatures during a warm season and high temperatures during a cold season had a protective effect on OHCA. The heat index followed a similar pattern, where an adverse effect was demonstrated for extreme levels of exposure.

**Conclusions:**

Evolving climate conditions characterized by excessive heat and low humidity represent risk factors for OHCA. As these conditions are easily avoided, by air conditioning and behavioral restrictions, necessary prevention measures are warranted.

**Supplementary Information:**

The online version contains supplementary material available at 10.1186/s12940-021-00722-1.

## Background

Out-of-Hospital Cardiac Arrest (OHCA) is defined as an absence of cardiac mechanical activity that occurs outside of a hospital setting. In 70–85% of cases the events have a cardiac etiology, e.g. myocardial infarction, congestive heart disease and heart rhythm change [1–3]. There are significant geographical variations in the incidence of and survival from OHCA. In a large systematic review, Asia was found to have the lowest incidence rate (52.5/100,000 person years), as compared to almost doubled incidence in North America (98.1/100,000 person years), Europe (81.6/ 100,000 person years), and Australia (112.5/100,000 person years) [4]. Approximately 382,800 adults in the U.S. experience an OHCA each year, over 90% of whom do not survive to hospital discharge [5–8]. Among those who survive to the Emergency Department, the long-term survival rates range between 3.0–16.3% [9–11].

Multiple studies have investigated the adverse effect of meteorological factors on health outcomes [1, 12–17]. In a recent Australian study, heatwaves were associated with significant increase in OHCA incidence. At a threshold of 95th percentile of yearly temperature, OHCA risk increased 1.25 times (95% CI 1.04; 1.50). When the heat threshold increased to 99th percentile, the relative risk increased to 1.48 (95% CI 1.11; 1.96) [17]. In a prospective study in Germany, Hensel et al. reported an increased likelihood of OHCA events for temperatures above 25 °C (17% increase) and below 5 °C (11% increase). Likewise, OHCA incidence increased by 33% below a threshold-value of 75% humidity compared to values above this cut-off [1]. The world’s heat-related excess deaths attributable to climate change are expected to be more than 90,000 deaths in 2030 and 255,000 deaths in 2050 [18].

In the past decade, the Middle East region has been subjected to heat waves, a prominent attribute of climate change phenomena, with an increasing incidence and severity [19]. Ground temperature in the area is expected to rise by 1 °C by the year 2050, and by up to 4 °C by the end of the current century [20] Similar trends have been registered in the Mediterranean basin [21].

The current investigation was performed in Israel located within the subtropical dryland zone (between 34.2 and 35.9°E and 29.5–33.4°N). Winter rains occur mainly during November through March, and the summer season is dry and hot. Despite its small size, Israel experiences sharp climatic and geographic gradients, both in the north-south and the east-west directions, covering three climate regions: a Mediterranean climate, a hot desert climate, and a narrow transitional strip of semi-arid climate [22].

The worsening climate conditions together with highly developed medical facilities and availability of the health data, make Israel a natural laboratory to study the effect of global warming on human health.

In this nationwide study, we aimed to investigate the association between the rate of out of hospital cardiac arrest and extreme meteorological conditions**.**

## Methods

We analyzed all the OHCA events treated by Magen David Adom (MDA) in Israel during 2016–2017. MDA is the national and largest Israeli Emergency Medicine Services (EMS) provider [23]. MDA responds to over 1000 emergency calls daily, whereas calls for OHCA account for 4 to 7% of all calls [23]. Nearly all cases of OHCA in Israel are treated by MDA staff [9]. MDA services are provided in twelve regional districts, each having a separate control center, which responds to the emergency calls, and directs the launch of ambulances [24].

The events were defined as OHCA by on-scene paramedics. Of note, the OHCA diagnosis in Israel made by EMS teams is not based on the Utstein templates. We excluded (i) cases of traumatic event causing OHCA, i.e. firearm wounds, stabbing, or motor vehicle accidents and (ii) OHCA cases in patients younger than 18 years. The information of the OHCA patients included the exact time and place of the event, survival to ED, age and gender. The time of the OHCA onset used in the study relied on the exact time the EMS team reported seeing the patient developing the condition or the time the emergency call was made to a dispatch center, if occurred prior to the EMS arrival.

### Exposure assessment

We used the climate data provided by the Ministry of Environmental Protection, based on monitored levels of temperature (°C), relative humidity (RH) (%) and solar radiation (SR) (W/m^2^) monitored on the half-hourly basis. These data have been validated by the Technion Center of Excellence in Exposure Science and Environmental Health (TCEEH) [25]. During the study period, there were overall 132 stations spread all over the entire Israel.

Additionally, we defined the heat index (HI) as a measure combining a simultaneous impact of heat and humidity on human health. The HI formula used in Israel [26, 27] is based on an arithmetical average between the dry bulb and the wet bulb temperature, resulting in a number known as “discomfort score”. To compute the wet-bulb temperature the following the formula was used:

$$ {T}_W= Tatan\left[0.151977{\left( RH\%+8.313659\right)}^{\raisebox{1ex}{$1$}\!\left/ \!\raisebox{-1ex}{$2$}\right.}\right]+\mathrm{atan}\left(T+ RH\%\right)-\mathrm{atan}\left( RH\%-1.676331\right)+0.00391838{\left( RH\%\right)}^{\raisebox{1ex}{$3$}\!\left/ \!\raisebox{-1ex}{$2$}\right.}\mathrm{atan}\left(0.023101 RH\%\right)-4.686035 $$

where: *T*− dry temperature, *T*_*w*_− wet temperature and *RH*− relative humidity.

We further computed the Discomfort score using the two types of temperatures, specifically, as *Discomfort score = [dry bulb temperature (°C) + wet bulb temperature (°C)] / 2.* The discomfort score, also known as the HI, is then grouped into groups of: < 22: no heat load, 22–23.9: mild heat load, 24–25.9: moderate heat load, 26–27.9: high heat load, 28–29.9: severe heat load, 30 and above: extreme heat load.

We used the seasonality division fitted for the eastern Mediterranean region, suggested by Alpert et al.: *summer* comprise the period of May 31st – September 22nd, *autumn* - September 23rd – December 6th, *winter* - December 6th – March 30th and *spring* - March 31st – May 30th [28].

Geocoding of the locations of the OHCA cases was performed using EZGeocode (ez34.net inc.) add-on for Google Sheets (Google Inc., USA). To add the elevation levels assessed for every OHCA location, we created a spatial joint between the XY locations of OHCA cases and elevation assessment using satellite-based Digital Elevation Model. The exposure assessment database was merged with the EMS records using the geocoded XY coordinates of the monitoring stations and OHCA locations. The exposure values were limited by the radius of 20 km and were based on the 5 monitoring stations that were the closest to the event. All environmental measurements were reported every 30 min and further averaged over 1 h.

To characterize a personal exposure in each OHCA case, we calculated a weighted average of exposure as measured by 5 monitors using an inverse of the squared distance from the case location to the monitor, i.e. Euclidian distance weighting assigning higher weight to closer monitors described by Hwang and Jaakkola [29]. The weights (*w*) of the ambient values were expressed by a function decreasing with distance to the monitoring station from the event location, specifically, $$ w=\raisebox{1ex}{$1$}\!\left/ \!\raisebox{-1ex}{${d}^p$}\right. $$, with *p* = 2, as most commonly used in this field, and d – representing the distance. When no measurements from monitoring stations were available within the 20 km radius of the OHCA event, those observations were excluded from the analysis.

We inspected all exposure variables for the possible exposure window associated with the outcome up to 72 h prior to the event. This exposure window is based on previous research by others reporting an effect seen for a lag of not more than 7 days prior to an event [30] and our preliminary findings of time-series analysis of the same data.

### Statistical analysis

We used a case-crossover approach, which inherently adjusts to all possible individual confounders. Specifically, the exposure within 72 h prior to the event (representing a case) was compared to exposure of the same subject 1 and 2 weeks before and 1 and 2 weeks after the event (representing a control), while the timing of the event at the control periods was identical to the exposure of the case period. We used exposure data also after the event, to avoid biased and/or confounded estimates resulting from a one directional case-crossover model [31, 32].

Continuous variables were expressed as mean ± standard deviation (SD), medians and ranges. Categorical variables were described by frequencies. Comparison of continuous variables was performed by t-test for normally distributed and Mann-Whitney test for variables for which normal distribution assumptions were not met; dichotomous variables were compared between study groups using Chi-Square or Fisher’s exact tests.

The exposure variables were presented by the 2-h moving average. The association between exposure and events was analyzed using conditional logistic regression in a lag-distributed non-linear model [33]. The lag matrix was defined by polynomial transformations with 3 degrees of freedom and exposure variables, i.e. temperature, humidity heat index and solar radiation, as piecewise-cubic splines with 3 knots evenly spread over the range. The choice of regression parameters was made based on the Akaike information criteria. The application of this methodology to the case-crossover setup is fairly recent [34] and reports on this particular usage have not been published so far. Odds ratio (OR) represented the main measure of an effect.

**Fig. 1:**
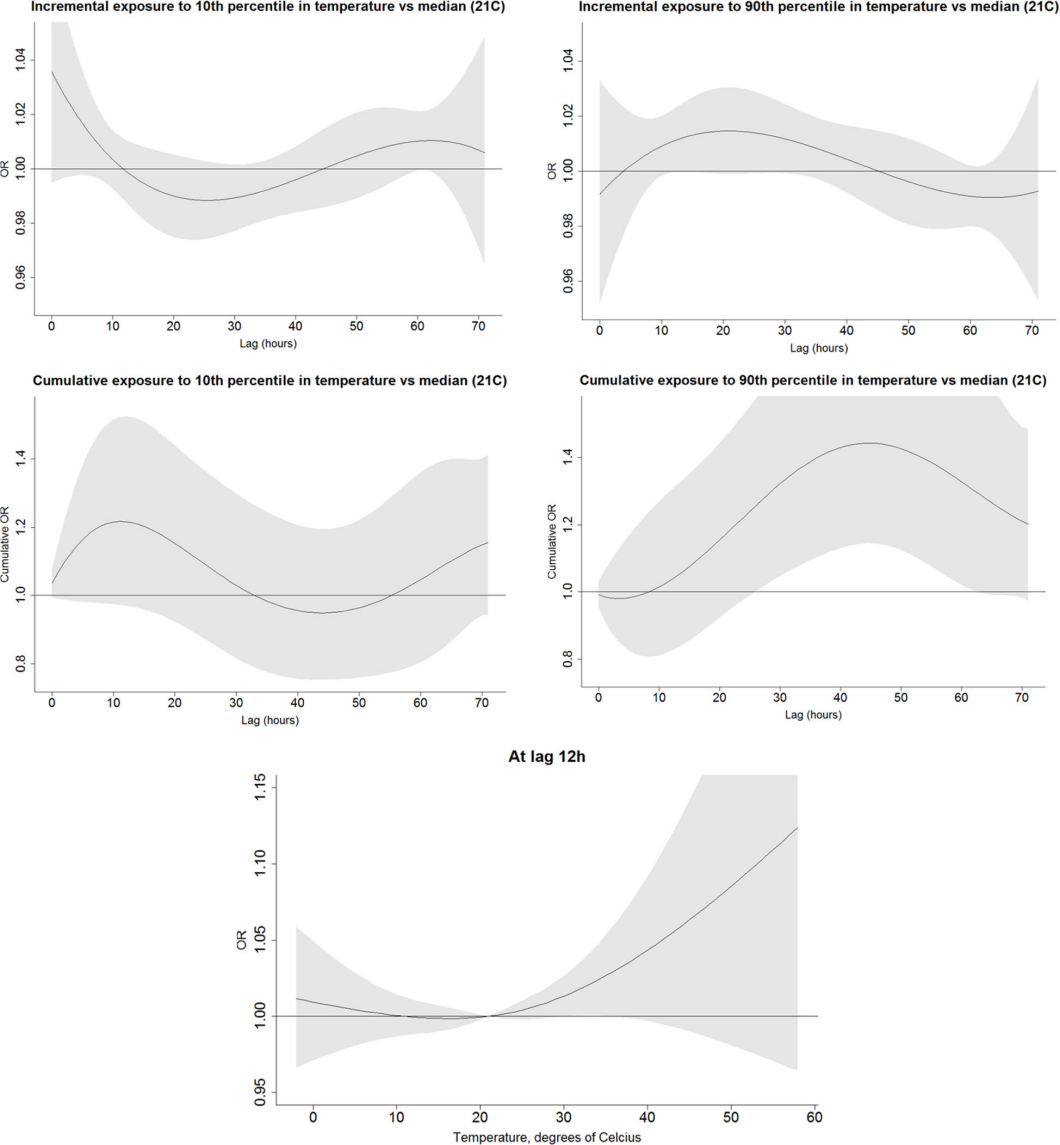
An association of temperature with OHCA events, adjusted to relative humidity. Figures in the upper row represent the incremental effect of exposure to temperature below the 10th percentile (11.5 °C) and above the 90th percentile (28.7 °C) as compared to the median value of 21 °C over the course of 72 h prior to the event onset. The figures in the second row show the cumulative effect of the respective exposures. The bottom figure represents an incremental effect of exposure to temperature at 12 h prior to the onset

**Fig. 2:**
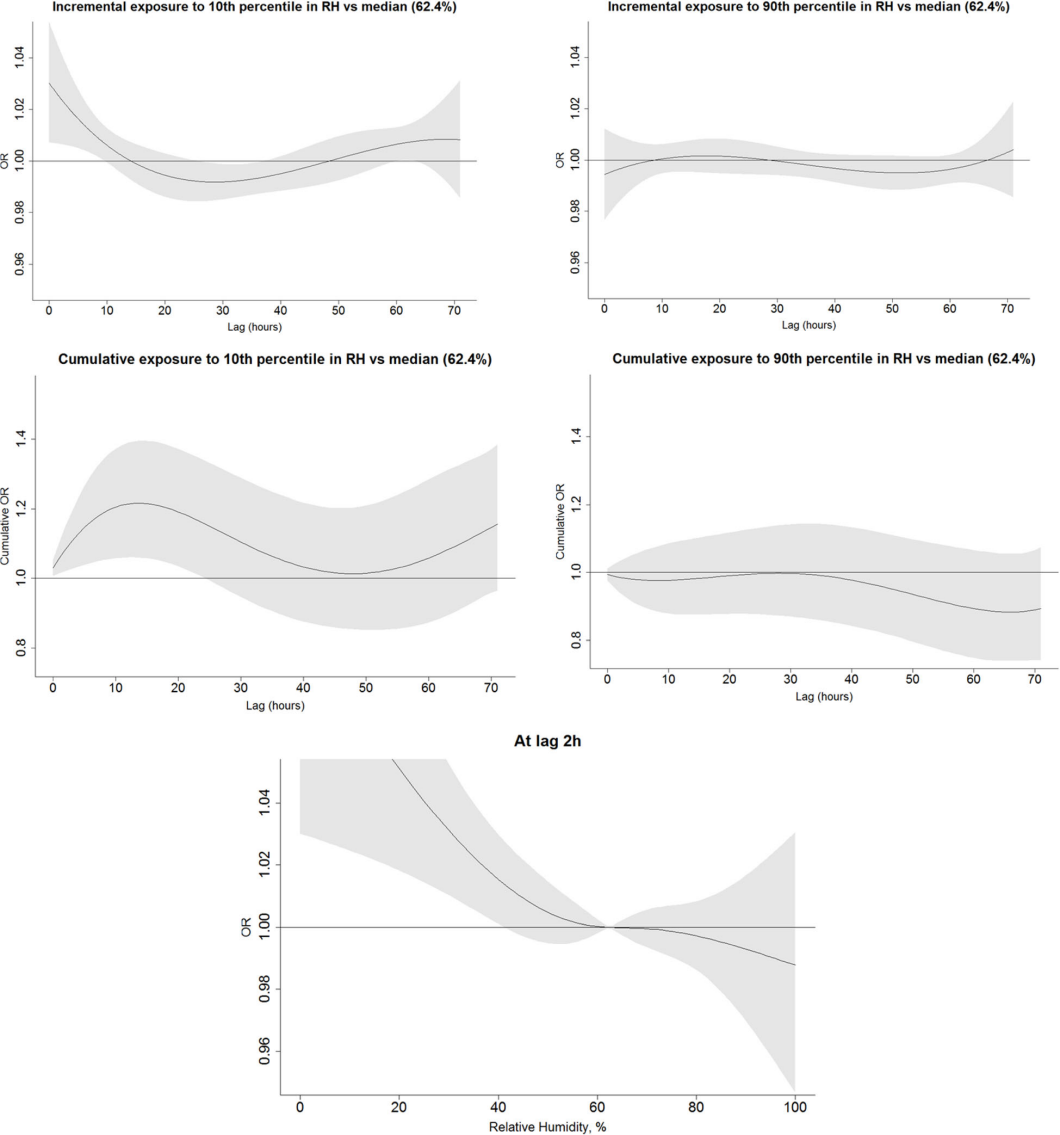
An association of relative humidity with OHCA events, adjusted to ambient temperature. Figures in the upper row represent the incremental effect of exposure to relative below the 10th percentile (33.9%) and above the 90th percentile (82.5%) as compared to the median value of 62.4% over the course of 72 h prior to the event onset. The figures in the second row show the cumulative effect of the respective exposures. The bottom figure represents an incremental effect of exposure to humidity at 2 h prior to the onset

**Fig. 3:**
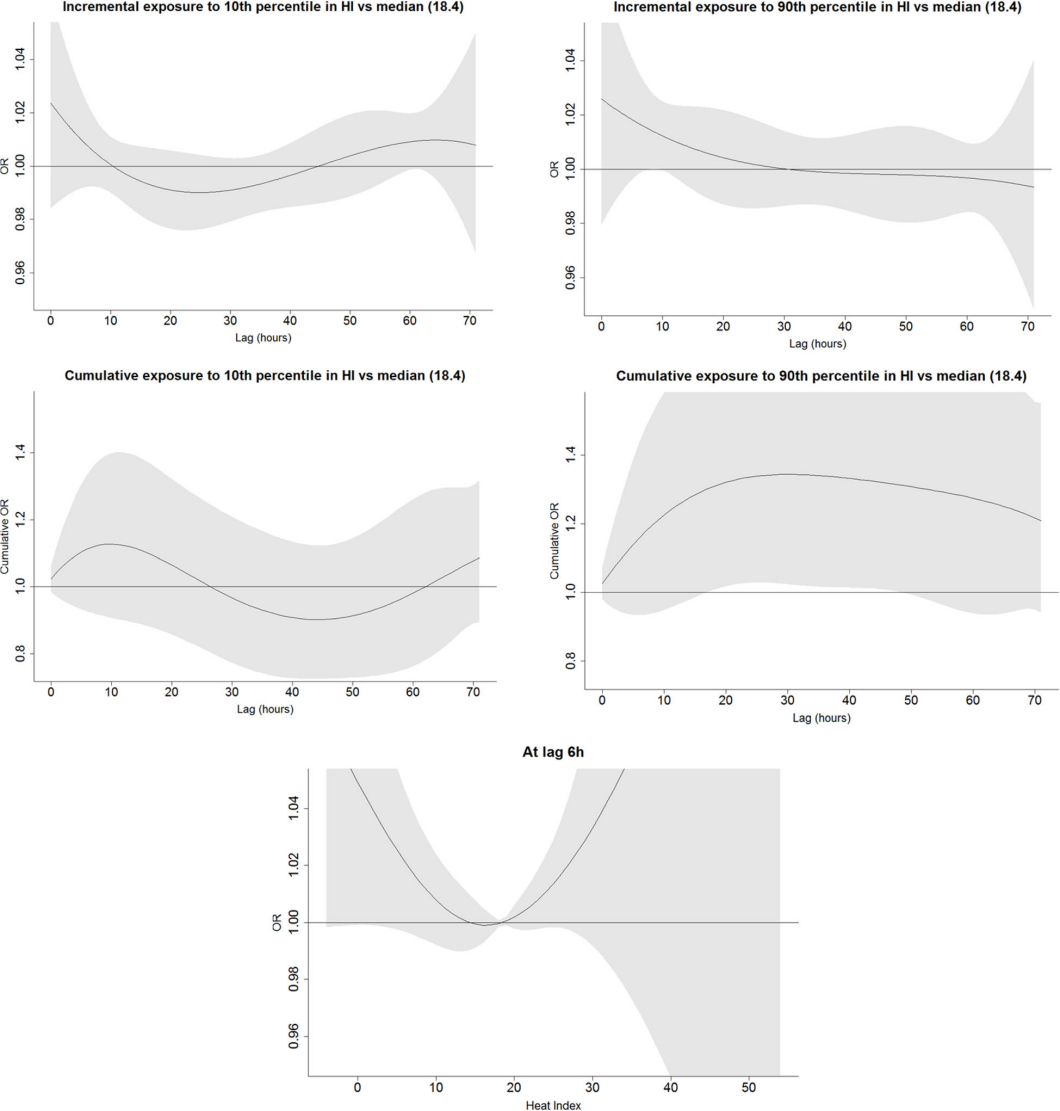
An association of heat index with OHCA events. Figures in the upper row represent the incremental effect of exposure to heat index below the 10th percentile (10) and above the 90th percentile (25.8) as compared to the median value of 18.4 over the course of 72 h prior to the event onset. The figures in the second row show the cumulative effect of the respective exposures. The bottom figure represents an incremental effect of exposure to heat index at 6 h prior to the onset

**Fig. 4:**
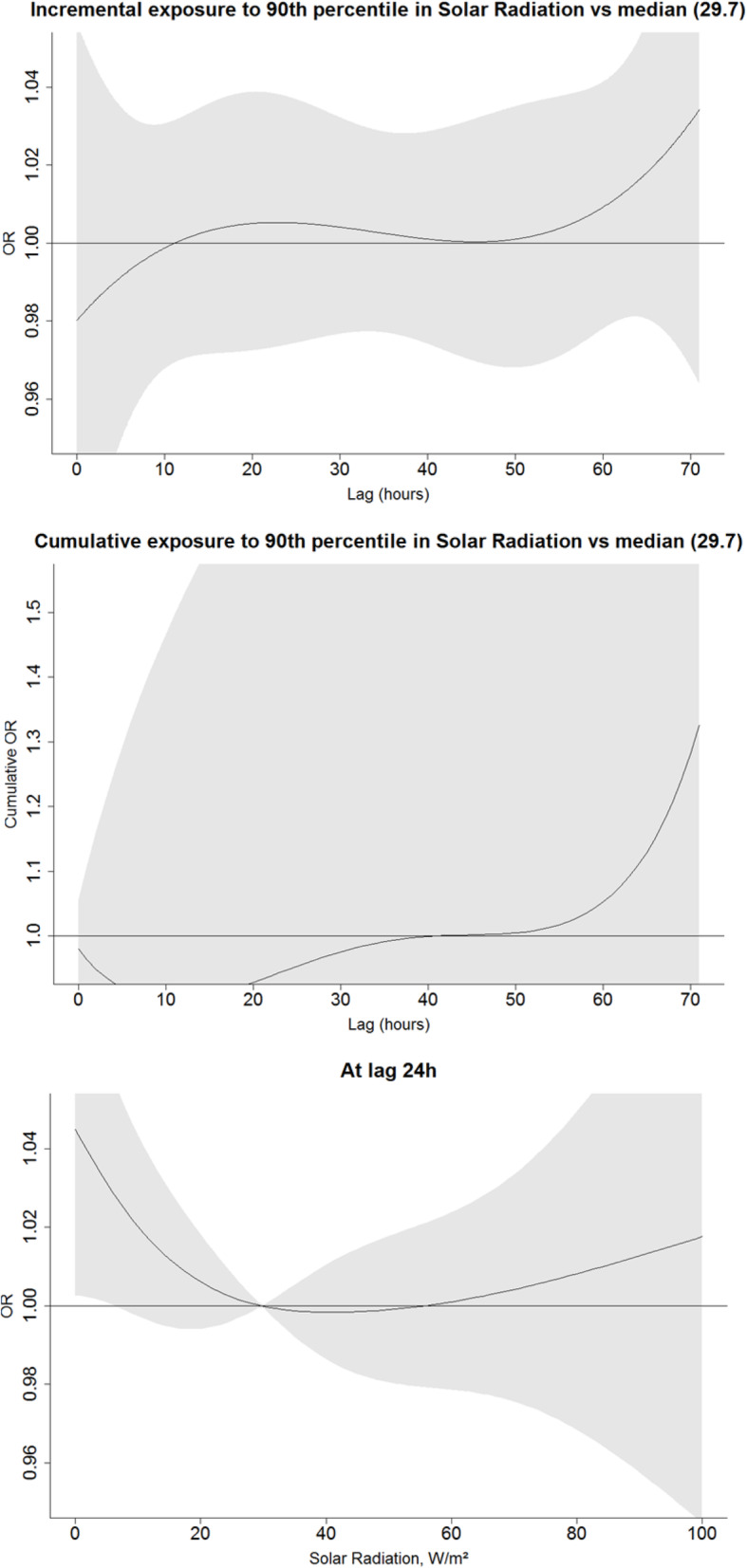
An association of solar radiation with OHCA events. Upper figure represents the incremental effect of exposure to solar radiation above the 90th percentile (73.2) as compared to the median value of 29.7 over the course of 72 h prior to the event onset. The figure in the middle shows the cumulative effect of the respective exposure. The bottom figure represents an incremental effect of exposure to solar radiation at 24 h prior to the onset

We explored the incremental and cumulative effects of temperature humidity and heat index by comparing the exposure to ≤10th and ≥ 90th percentile over the period of 72 h prior to the event or a reference period to the median value of the respective factor (Figs. 1, 2, and 3). As opposed to the U-shape relationship assumed for heat and humidity, only the excessive levels of exposure to solar radiation were explored (Fig. 4). The analyses were summarized by an exposure-outcome curve at a selected exposure window suggested by the findings from the previous steps.

We explored the possibility of mutual confounding between the meteorological factors by investigating the difference between crude and adjusted point estimates of an association. A deviation of 10% between the two indicated a possibility of confounding, in which case the adjustment was performed throughout the entire analysis in attempt of presenting an independent contribution of each factor.

We performed a subgroup analyses by gender, age (within a group of 70+), dead-on-scene or alive status of the subjects, day of a week and daytime vs. night. In the later analysis, we considered 7 am-7 pm as a day time and the 7 pm-7 am to be a night time. To address the diverse climate in Israel we chose to explore three different metropolis areas, featured by relatively high number of events, i.e. Tel-Aviv city and its neighborhood, Jerusalem and Haifa.

The data were analyzed using SAS9.4 and R statistical software (package “dlnm”), version 4.0.2. Statistical significance was set at *p*-value< 0.05.

## Results

During the study period of 2016–2017 the EMS services in Israel received 12,401 calls related to OHCA. The average age of the study population was 75.5 years, majority of them being males (55.8%), and only one third of them survived to the arrival of the EMS teams (Table 1). The OHCA weekly incidence rate was evenly spread over the seasons, as well as over the week, with 28.7% occurring during the two days of the weekend. Over 60% of OHCA calls were made during the daylight hours.

**Table 1:** Demographical characteristics of the OHCA patients in Israel and geographical and temporal features of events, Israel 2016–201**7**

	***N*** = 12,401 patients
Age, years	
Mean ± SD (n)	75.5 ± 16.2 (12,401)
Median	79.1
Min; Max	18.1; 112.3
Age grouped, % (n/N)	
18–45	5.4 (674/12401)
45–65	17.7 (2190/12401)
65–85	42.1 (5223/12401)
85+	34.8 (4314/12401)
Male gender, % (n/N)	55.8 (6919/12401)
Dead on scene, % (n/N)	68.3 (8470/12401)
Elevation, meters	
Mean ± SD (n)	165.2 ± 238.42 (12,401)
Median	54.0
Min; Max	−382.0; 1153.0
Season, % (n/N)	
Summer *(May 31 – Sep 22)*	29.1 (3609/12401)
Fall *(Sep 23 – Dec 6)*	21.7 (2687/12401)
Winter *(Dec 7 –Mar 30)*	33.5 (4152/12401)
Spring *(Mar 31 – May 30)*	15.7 (1953/12401)
Weekend, % (n/N)	28.7 (3562/12,401)
Day shift (7 am-7 pm), % (n/N)	61.6 (7639/12,401)

Table 2 shows the ambient values measured during the time of the 12,401 cases and 49,604 control periods. The average hourly temperatures ranged from 0 °C to 43 °C, with median at 21.0 °C, and 10th and 90th percentile at 11.5 °C and 28.7 °C. Relative humidity measured in the study ranged between 3.0–99.0%, with 10% of the observations below 33.9 and 90% above 82.5%. The records of solar radiation were available in 40,445 cases, and ranged between 0 and 99.0 W/m^2^. Temperature and solar radiation were strongly and positively correlated between each other (Spearman rho = 0.43, *p*-value< 0.001) and negatively correlated with the relative humidity (Spearman rho = − 0.32, p-value< 0.001).

**Table 2:** Environmental Exposures, on the day of the event or the control period (*N* = 62,005 days)

Meteorological conditions	
**Temperature,** ^**0**^**C**	
Mean ± SD (n)	20.5 ± 6.5 (50064)
Median	21.0
Min; Max	0.0; 43.0
IQR	10.5
10th; 90th percentile	11.5; 28.7
**Relative Humidity,** ^**%**^	
Mean ± SD (n)	60.1 ± 18.4 (56547)
Median	62.4
Min; Max	3.0; 99.0
IQR	23.9
10th; 90th percentile	33.9; 82.5
**Heat Index, discomfort units**	
Mean ± SD (n)	18.4 ± 5.9 (50064)
Median	18.4
Min; Max	−3.5; 37.5
IQR	9.8
10th; 90th percentile	10.0; 25.8
**Solar Radiation, W/m**^**2**^	
Mean ± SD (n)	33.1 ± 26.7 (40445)
Median	29.7
Min; Max	0.0; 99.0
IQR	45.1
10th; 90th percentile	1.5; 73.2
**Wind Speed, m/s**	
Mean ± SD (n)	1.8 ± 1.3 (57483)
Median	1.5
Min; Max	0.0; 17.4
IQR	1.6
10th; 90th percentile	0.5; 3.5

We explored the possibility of confounding between the factors (supplementary materials, Table 1). Based on the analysis, to segregate the possible independent contribution of relative humidity to OHCA events, it should be adjusted to either temperature or solar radiation (temperature was chosen). Likewise, to estimate an independent effect of temperature and solar radiation, both have to be adjusted to relative humidity. Since heat index is a function of relative humidity and temperature, this factor could be analyzed as crude. These findings justified the set of adjustment covariates used throughout the analysis.

**Fig. 5:**
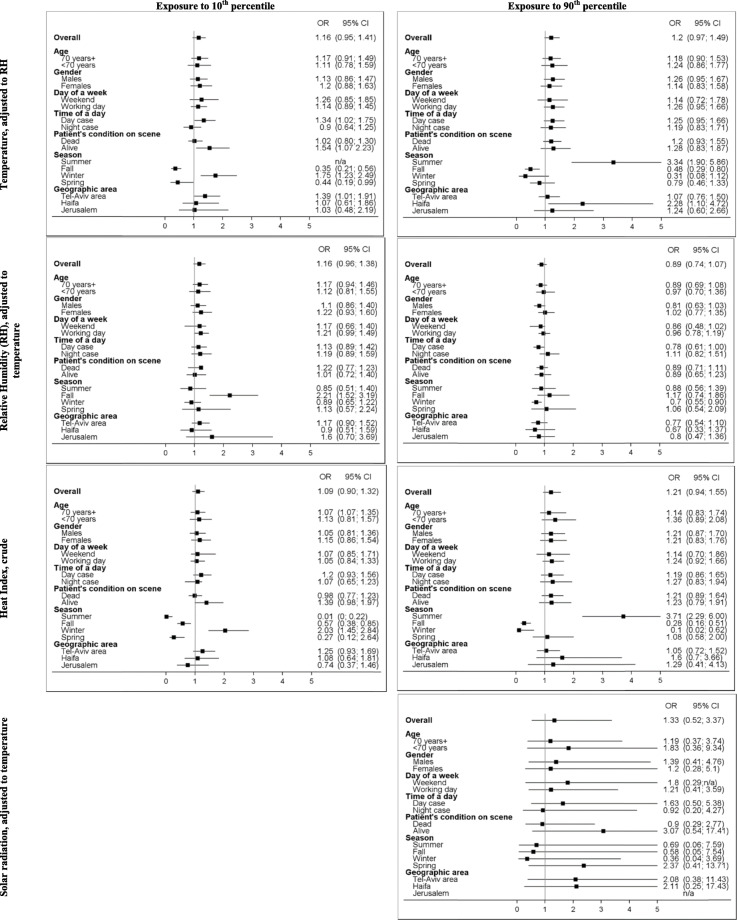
A cumulative effect of exposure to various meteorological conditions as compared to median value. Subgroup analyses by demographical, clinical, temporal and geographical factors

The exposure to excessive outdoor average daily temperature (>90th percentile) adjusted to relative humidity was adversely associated with OHCA during the 10–20 h prior to the event as compared to the median temperature of 21 °C. Furthermore, the cumulative impact of exposure to 90th percentile of temperature over the 3 days prior to the event expressed in OR was equal 1.2 although with borderline significance, 95%CI 0.97; 1.49 (Fig. 5). Exposure to excessively high temperatures 30.0–40.0 °C measured at 12 h prior to an event was positively associated with OHCA. For instance, an exposure to 35 °C was associated with an OR 1.02 (95%CI 1.00; 1.05) as compared to the median 21 °C. Exposure to weather colder than median was relevant just few hours prior to an OHCA event and its cumulative effect over the 72 h was associated with an OR = 1.16 and borderline significance, 95%CI 0.95; 1.41 (Fig. 5).

Exposure to humidity at 90th percentile independent of temperature was not associated with an increase in OHCA, whereas exposure to extremely dry conditions (relative humidity ≤10th percentile, 34%) measured within 10 h prior was positively associated with the event (Fig. 2). The cumulative exposure to humidity below 10th percentile over the period of 72 h prior to an event, independent of ambient temperature, were adversely associated with OHCA although with a borderline significance (OR 1.16, 95%CI 0.96; 1.38). The close inspection of the association between humidity measured 2 h prior to an event indicated that values below 40% were the most detrimental to the risk of OHCA events (with OR in the range 1.01–1.04 for humidity values 0–40% as compared to the median 62.4%) (Fig. 2).

Heat index above 90th percentile, reflecting the discomfort burden based on the temperature and humidity, was associated with the OHCA occurrence at a borderline level of significance (OR = 1.21, 95%CI 0.94; 1.55) when compared to its median. This exposure reached the statistical significance when accumulated over the 20 h prior to the event (Fig. 3).

Exposure to the ≥90th percentile of solar radiation did not increase the risk (Figs. 4 and 5). Elevation did not alter risk estimates of other exposures.

The subgroup analysis showed heterogeneity in effects especially in temporal and geographical subgroups (Fig. 5). An estimate of an association between OHCA and exposure to extremely high temperatures independent of humidity levels was not dramatically different between sexes and age. However, a cumulative exposure to temperatures above the 90th percentile were associated with OHCA if experienced in summer (OR = 3.24, 95%CI 1.90; 5.86) and if measured in Haifa area (OR = 2.28, 95%CI 1.10; 4.72). Exposure to temperatures below the 10th percentile produced an adverse association with OHCA within subjects found alive (OR = 1.54, 95%CI 1.07; 2.23) as compared to subjects dead on-scene (OR = 4.02; 0.80; 1.30), if measured in winter (OR = 1.75, 95%CI 1.23; 2.49) and / or if occurred in Tel-Aviv area (OR = 1.39, 95%CI 1.01; 1.91). The exposure to extreme hot and cold temperatures also had a protective effect. Specifically, the ORs for temperatures above 90th percentiles were 0.48 (0.29; 0.80) and 0.31 (95% 0.08; 1.12) for fall and winter, respectively. The ORs for temperatures below 10th percentile were 0.35 (95%CI 0.21; 0.56) and 0.44 (0.19; 0.99) for fall and spring, respectively. No temperatures below the 10th percentile were recorded in summer. The pattern of adverse effect for excessive values in summer and low values in winter, as well as the protective effect for opposing conditions, was repeated for the heat index (Fig. 5).

Dry conditions (relative humidity ≤10th percentile) independent of temperature levels did not vary by demographic characteristics of the patients. An adverse association of low humidity with OHCA was recorded for the cases in fall (OR = 2.21 95%CI 1.52; 3.19).

The subgroup analysis of solar radiation adjusted to temperature did not indicate any variation of an effect between the sub-groups.

## Discussion

The current study assessed Out-Of-Hospital-Cardiac-Arrest events on a national level over a period of two years. Our analysis provides an evidence of an adverse association between harsh meteorological conditions and OHCA events. Specifically, we found that extremely high temperature and dry conditions were likely to occur within 72 h prior to an OHCA event.

The results of the current analysis can be projected onto future meteorological conditions in the Middle East and used to model the implications of the climate change on public health in the region. The 90th percentile set in our study to 29 °C is an actual value that is far from being extreme in many European cities in summer.

High temperature has been repeatedly shown to be associated with mortality [12, 35], and particularly with OHCA [1, 36–40]. Our findings support the connection, stressing the importance of the 12–20 h interval prior to the event and temperatures above 30 °C as compared to the local median of 21 °C. The effect of heat on OHCA is widely believed to be driven by dehydration [41, 42]. Some suggest that heat is associated with a vasodilation of peripheral blood vessels, resulting in reduced coronary blood flow, leading to arrhythmias and heart failure [36].

Relatively cold temperatures, have been shown to present a risk factor for mortality [43] and specifically for OHCA [1, 37, 44]. This is consistent with our findings, pointing at the few hours prior to the event as the most vulnerable with respect to extremely cold temperatures as compared to the local median of 21 °C.

Of note, the extreme temperatures both low and high, had an adverse effect if experienced during the respective cold or hot season. Consequently, low temperatures during warm season and high temperatures during cold season had a protective effect. In other words, the extremity of exposure in both directions of the range in relation to the background temperatures was shown to be most relevant for OHCA occurrence, rather than their absolute change.

We recorded a negative association with humidity, independent of temperature levels, although with borderline significance (95%CI (OR) 0.96; 1.38). This is opposed to previously reported positive association, whereas OHCA cases were more likely to be exposed to higher humidity conditions [45]. On the contrary, our findings point at the possibility that extreme dry conditions might present a considerable risk factor for OHCA event. Similar findings have been reported in Burkina Faso, whereas particularly hot and dry season (March–May) was featured by elevated cardiovascular mortality rates [46]. A panel study of 16 subjects recorded the physiological and subjective responses to different levels of humidity. Although, low humidity had no effect on blood pressure or heart rate, relative humidity levels below 10% were associated with dryness of the nasal mucous membrane, eyes and skin [47]. Researchers in another panel study of 14 males hypothesized that physiological parameters, like body temperature or heat rate, vary under hot and dry conditions and this variation is modified by age; however, no substantial differences were found [48]. Thus, the pathological mechanism of low humidity on cardiovascular events is not well established. In general, panel studies analyzing an impact of heat and humidity in elderly are scarce and underpowered. This warrants a more extensive research of an aging population, especially under the threat of the global warming.

Our study was underpowered to detect effect modification by demographic factors when adjustments to other meteorological factor were performed.

The analysis *benefited* from the nationwide format of the exposure and events databases that ensures minimal selection bias in our conclusions. Specifically, the exposure assessment was provided by the net of monitoring stations spread all over the country and events – recorded by the national EMS services. Moreover, records in both databases were reported at a minimal resolution of 30 min. This enabled a valid time-specific analysis of exposure-event link, critical for causal interpretation of the findings.

Our study has a number of *limitations.* There is a possibility of misclassification bias, since EMS data included only a location where an event occurred, whereas, the residence address was not available to the researchers and the patient may have not been at the event location for the entire exposure window studied. On the other hand, we believe this issue may have caused bias towards the null hypothesis.

Furthermore, the subjects with an event in remote areas might have sparse or no monitoring stations at all within 20 km of their event location. This could potentially result in an exposure estimation based on fewer monitoring stations (misclassification bias) when former occurred or selection bias – when latter.

Additionally, the background morbidity of the patients was not available from the database. However, the case-crossover design helped to control for all prior medical conditions, since each case served a control for him or herself. Furthermore, there is a chance of non-cardiac deaths mistakenly included in the analysis, although their portion might be negligible given that all apparent cases of a non-cardiac death have been excluded from the analysis.

## Conclusions

The evolving climate conditions characterized by the excessive heat and low humidity represent risk factors for OHCA. As these conditions are easily avoided, by air conditioning and behavioral restrictions, necessary prevention measures are warranted within the vulnerable subjects.

## Supplementary Information

**Additional file 1: Table 1**. Exploration of associations between the meteorological factors.

## Data Availability

The data that support the findings of this study are available from the Clinical Research Center of the Soroka University Medical Center (Beer-Sheva, Israel), but restrictions apply to the availability of these data, which were used under IRB approval for the current study, and so are not publicly available. Data are however available from the authors upon reasonable request and with permission of the IRB committee of the Soroka University Medical Center.

## Abbreviations

EMS: Emergency Medical Services
HI: Heat Index
MDA: Magen David Adom
OHCA: Out-of-hospital-cardiac arrest
OR: Odds Ratio
RH: Relative Humidity
SR: Solar Radiation
