# Supplementary material for: Extreme temperature and out-of-hospital-cardiac-arrest. Nationwide study in a hot climate country
Source: Environ Health. 2021 Apr 5;20:38. doi: 10.1186/s12940-021-00722-1 (PMC8022396; doi:10.1186/s12940-021-00722-1)
Supplement: Supplementary file 1 — Additional file 1: Table 1. Exploration of associations between the meteorological factors. [file 12940_2021_722_MOESM1_ESM.docx]

# Supplementary material

# Table 1: Exploration of associations between the meteorological factors

| **Environmental Factor** | **Association type** | **Cumulative effect as compared to median** | | | |
| --- | --- | --- | --- | --- | --- |
|  |  | **Exposure to 10^th^ percentile** | | **Exposure to 90^th^ percentile** | |
|  |  | **OR** | **95%CI (OR)** | **OR** | **95%CI (OR)** |
| Temperature (**Temp**) | **Crude** | **1.05** | **0.87; 1.27** | **1.37** | **1.13; 1.66** |
|  | Adjusted to RH | 1.16 | 0.94; 1.41 | 1.20 | 0.97; 1.49 |
|  | Adjusted to SR | 0.98 | 0.73; 1.32 | 1.10 | 0.80; 1.53 |
|  | Adjusted to RH & SR | 0.98 | 0.72; 1.34 | 1.02 | 0.72; 1.44 |
| Relative Humidity (**RH**) | **Crude** | **1.20** | **1.07; 1.36** | **1.03** | **0.90; 1.17** |
|  | Adjusted to Temp | 1.16 | 0.96; 1.38 | 0.89 | 0.74; 1.07 |
|  | Adjusted to SR | 1.12 | 0.94; 1.33 | 1.04 | 0.86; 1.24 |
|  | Adjusted to Temp & SR | 1.14 | 0.87; 1.49 | 0.94 | 0.72; 1.22 |
| Solar Radiation (**SR**) | **Crude** |  |  | **1.74** | **0.96; 3.14** |
|  | Adjusted to Temp |  |  | 1.32 | 0.52; 3.37 |
|  | Adjusted to RH |  |  | 1.60 | 0.86; 2.99 |
|  | Adjusted to Temp & RH |  |  | 1.36 | 0.53; 3.49 |
| Heat Index (**HI**) | **Crude** | **1.09** | **0.90; 1.32** | **0.78** | **0.63; 0.97** |
|  | Adjusted to SR | 1.01 | 0.75; 1.35 | 0.93 | 0.65; 1.34 |
